# Detection of exon skipping events in *BRCA1* RNA using MLPA kit P002

**DOI:** 10.1007/s11033-012-1575-2

**Published:** 2012-02-17

**Authors:** Rita D. Brandão, Demis Tserpelis, Encarna Gómez García, Marinus J. Blok

**Affiliations:** 1 Department of Clinical Genetics, Maastricht University Medical Centre, P.O. Box 616, 6200 MD Maastricht, The Netherlands; 2 Department of Clinical Genetics, Maastricht University Medical Centre, P.O. Box 5800, 6202 AZ Maastricht, The Netherlands; 3 GROW—School for Oncology and Developmental Biology, Maastricht University Medical Centre, Maastricht, The Netherlands

**Keywords:** RNA splicing, RNA, Multiplex ligation probe amplification (MLPA), Breast cancer, *BRCA1*

## Abstract

A rapid and easy method to screen for aberrant cDNA would be a very useful diagnostic tool in genetics since a fraction of the DNA variants found affect RNA splicing. The currently used RT-PCR methods require new primer combinations to study each variant that might affect splicing. Since MLPA is routinely used to detect large genomic deletions and successfully detected exon skipping events in Duchenne muscular dystrophy in cDNA, we performed a pilot study to evaluate its value for* BRCA1 *cDNA. The effect of puromycin, DNase I and two different DNA cleaning protocols were tested in the RNA analysis of lymphocyte cultures. We used two samples from unrelated families with two different *BRCA1* exon deletion events, two healthy unrelated controls and six samples from hereditary breast/ovarian cancer syndrome (HBOC) patients without *BRCA1*/*2* mutations. Using RNA treated with DNase I and cleaned in a column system from puromycin-treated fractions, we were able to identify the two *BRCA1* deletions. Additional HBOC patients did not show additional splice events. However, we were not able to get reproducible results. Therefore, the cDNA-MLPA technique using kit *BRCA1* P002 is in our hands currently not reliable enough for routine RNA analysis and needs further optimization.

## Introduction

Genetic screening of the *BRCA1* and *BRCA2* genes is offered to families with high risk of breast and ovarian cancer. Besides clear pathogenic mutations and polymorphisms, unclassified variants (UVs) of unclear clinical relevance are found. Some of these UVs may result in aberrant splicing, by affecting the donor or acceptor splice sites, or exonic splice site enhancer (ESE) sites [1] as predicted *in silico*. Additionally, deep intronic variants, which are normally ignored, may also affect splicing. One example of a deep intronic pathogenic variant is the variant CDKN2A IVS2-105A>G, which causes retention of intronic sequence [2]. Another example is the mutation c.903+409T>C in the MTRR (methionine synthase reductase) gene, which activates a pseudoexon, causing a frameshift insertion that leads to a premature stop codon [3]. Experimental proof is needed to confirm the predicted changes in RNA splicing. The experiments are usually performed using RT-PCR, for which a set of specific primers targeted to the relevant cDNA region is needed for every new variant [4–7]. It is noteworthy that exon skipping is the most common alternative splice event [8]. After the report of Kesari et al. [9], who were able to detect skipping events on cDNA from the Duchenne muscular dystrophy (DMD) gene using the respective genomic multiplex ligation probe amplification (MLPA) kit, we sought to evaluate the use of a commercially available *BRCA1* MLPA kit [10] for the detection of exon skipping in cDNA instead of genomic DNA. *BRCA1* MLPA is a multiplex assay based on the hybridization of a large set of primers throughout the entire coding part of the *BRCA1* gene. Therefore the assay should potentially also be able to detect all exon skipping events in cDNA in the presence of a variant affecting splicing, without the need to design a specific RT-PCR assay for each variant. Although these are likely rare events, using a rapid and relatively cheap assay to assess them would be valuable in a diagnostic setting to rule out their presence.

For this pilot study, samples with *BRCA1* exon 13 skipping (c.4242-1643del3835) or exon 22 skipping (c.5333-36del510) [11] were selected. The study also included samples from two unrelated healthy controls and six samples from patients belonging to high risk families for which no *BRCA1* or *BRCA2* mutation was identified in the standard diagnostic screening. Here we show that the MLPA method was able to detect the skipping events, but it was not reproducible enough for use in clinical testing despite the optimization attempts which are here described.

## Materials and methods

### Cell culture

White blood cells were isolated and cultured in complete medium consisting of: RPMI 1640 supplemented with l-glutamine (Gibco) and 12.5% FCS with additional supplements and antibiotics. Lymphocyte growth was stimulated with 50 μL/mL PHA (Gibco) and 10 U/mL of IL-2 (Roche). At day 7, 4–6 h before harvesting the cells, cultures were treated with 200 μg/mL of puromycin (Sigma), to enrich for transcripts containing premature stop codons by the inhibition of NMD [12].

### RNA isolation, cDNA synthesis and MLPA reaction

Total RNA was isolated using TRIzol (Invitrogen) or TRIpure (Roche) reagent. RNA samples used were either not subjected to DNase I treatment or treated with DNA-free kit (AMBION) or with DNase I treatment followed by purification in the column system RNeasy MinElute Kit (Qiagen). First-strand cDNA was obtained with Reverse Transcriptase M-MUL (Finnzymes) using random hexamers (Invitrogen) following the manufacturers’ instructions. The cDNA was amplified with the SALSA MLPA P002 probe mix (MRC-Holland) according to the manufacturer’s protocol. Fragment analysis was performed by capillary electrophoresis in an ABI PRISM 3730 automatic sequencer (Applied Biosystems).

### Data analysis

The size calling and the peak areas were assessed using the Genemarker software (Softgenetics) and exported to a “.txt” file. The values of the antisense probes were extremely low compared to the sense probes, and they do not have known biological meaning. Therefore, the data was filtered to leave only the data from probes corresponding in sequence to that of sense *BRCA1* mRNA. The normalization of the data was performed using a spreadsheet according to the Manual spreadsheet-based MLPA analysis instructions (available on the MRC-Holland website: www.MLPA.com). The threshold values for deletions and duplications were set to 0.75–1.25, respectively, which are also used for DNA analysis [13–16].

## Results

With the SALSA MLPA P002 kit, strong signals were obtained for 21 out of 25 probes. These probes contained more than 85% nucleotides hybridizing to the exon sequence in the correct orientation. The signals for the probes with less than 85% matching exonic sequence (exons 1A, 9 and 19) or in antisense (23) were extremely weak and often not even detectable by the software. This also confirms the absence of contaminating genomic DNA in the RNA samples.

**Fig. 1:**
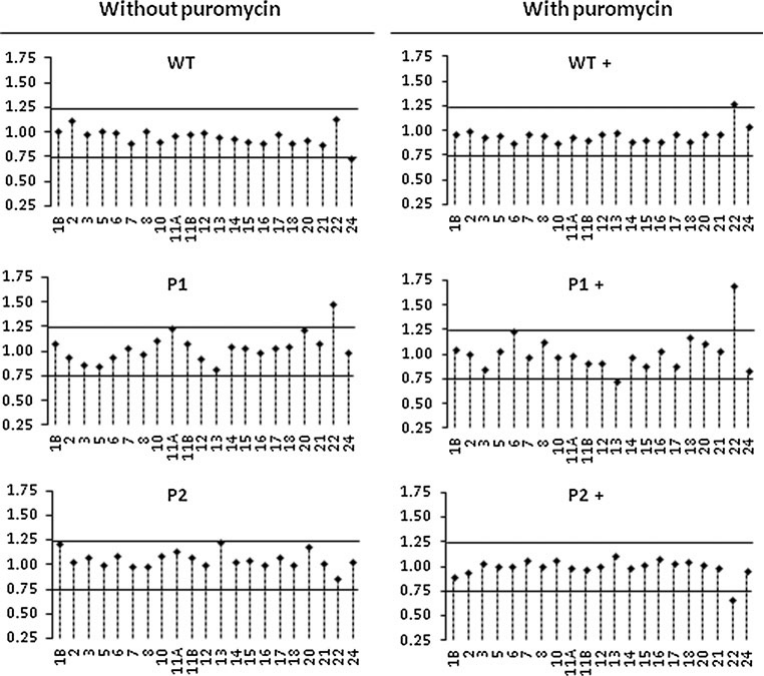
MLPA results obtained using puromycin-treated and non-treated samples as indicated. Healthy controls without* BRCA1 *mutations are indicated as *WT*, whereas *P1* and *P2* are positive controls with exon 13 and exon 22 deletion events, respectively

Initially, we have compared the results from puromycin-treated and non-treated samples (Fig. 1), without DNase I treatment. The results were not optimal, but it was observed that the puromycin-treated samples gave better results than the non-treated. Subsequently, we tested the effect of two different DNase I treatment options: (1) DNase I treatment followed by purification in a column system and (2) DNase I treatment kit that allows to remove the enzyme by precipitation and centrifugation. The results were considerably improved when the RNAs were cleaned in a column system (data not shown), i.e. variation in the signals among individuals was greatly reduced, at least in two independent experiments.

**Fig. 2:**
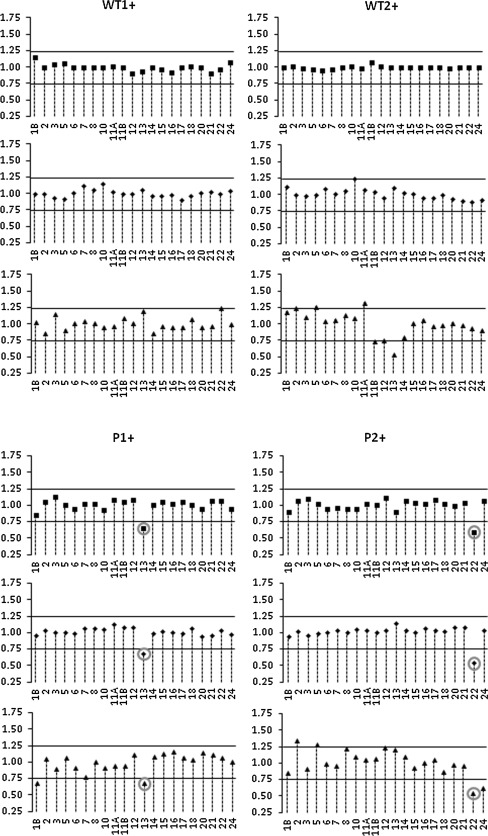
MLPA results obtained in three independent experiments for puromycin-treated fractions. Healthy controls without* BRCA1 *mutations are indicated as *WT*; *P1* and *P2* are positive controls with exon 13 and exon 22 deletion events, respectively

Six samples from high risk families without a *BRCA1*/*2* mutation were also analyzed (data not shown) using the puromycin-treated fractions and RNAs treated with DNase I and cleaned in a column system. None of these samples showed an exon skipping event, in the 20 exons tested. However, in an independent third experiment we observed increased interindividual variability in some exon signals. Many exons had normalized values outside the 0.75–1.25 thresholds (Fig. 2). This was also observed in healthy control samples. This hampers the evaluation of splicing defects as it suggests duplications or deletions events that would need experimental follow-up or repetitive MLPA analysis to determine reproducibility.

## Discussion

The MLPA method is widely used in diagnostics, mainly to test genomic events such as deletions and duplications. Although there are a few commercial RT-MLPA kits, these are designed to test the expression of genes associated with certain biological processes, MRC-Holland has not developed RT-MLPA kits to test splice events. Besides the use of the MLPA, or other multiplex approaches, to test the effect of genetic variants predicted to affect splicing at the RNA level, it would be useful to test for *BRCA1* and *BRCA2* mutation negative patients with strong breast and/or ovarian cancer history. This group of patients may carry variants outside the screened intronic region flanking the exons which could affect splicing. Since exon skipping is the most common alternative splice event [8], developing a test that allows to screen for exon skipping events would detect the majority of alternative splice events.

One single study has previously shown that MLPA could be used to test exon skipping events in RNA transcripts of the *DMD* gene [9]. Here we report the use of MLPA kit for the analysis of* BRCA1 *exon skipping events. The most optimal results were obtained from puromycin-treated samples and when RNA was treated with DNase I and subsequently purified in a column system. However, despite efforts to optimize the technique further, we were not able to get reliable, reproducible results for unequivocal interpretation using the kit* BRCA1 *P002. This variation was also observed in healthy control samples, which showed both deletion and duplication events in one out of three experiments performed.

MLPA test is a flexible multiplex assay which allows for up to a total of 50 probes and in principle, it should be possible to use it for detection of alternative splicing events other than exon skipping. To be able to test also for intron retention or insertion of pseudoexons, probes crossing over exon–exon boundaries should also be included in the assay. Although mRNA-seq technology [17] will also allow to test for aberrant splicing events in patients, MLPA could be a more cost-effective technique. However, it needs to be optimized further for routine use.
